# No Two Systems Are the Same: Paramedic Perceptions of Contemporary System Performance Using Prehospital Quality Indicators

**DOI:** 10.7759/cureus.35859

**Published:** 2023-03-07

**Authors:** Timothy Makrides, Ford Smith, Linda Ross, Cameron M Gosling, Joseph Acker, Peter O'Meara

**Affiliations:** 1 Department of Paramedicine, Monash University, Melbourne, AUS; 2 Clinical Hub, BC Emergency Health Services, Vancouver, CAN; 3 Faculty of Medicine, University of British Columbia, Vancouver, CAN

**Keywords:** performance, comparison, prehospital quality indicator, system, ems, ambulance, paramedic

## Abstract

Introduction

In recent years, researchers have identified two new models of paramedicine within the Anglo-American paramedic system known as the Directive and Professionally Autonomous paramedic systems. The research team now seek to compare paramedic perception of system performance between the two systems using prehospital quality indicators.

Methods

Paramedics employed within Anglo-American paramedic systems undertook a survey evaluating their experience and perception of system performance against a set of modified prehospital quality indicators. Data were collected using a survey combining single-choice questions with matrix multiple-choice questions. Key results were cross-tabulated with demographic (informant) and system factors to compare performance between the two new paramedic systems.

Results

The survey indicated a substantial difference in perceived clinical and operational performance between the Professionally Autonomous and Directive paramedic systems, with the Professionally Autonomous paramedic system performing consistently better in all 11 prehospital quality indicator domains.

Conclusion

The results of this survey are a vital step in helping paramedics, health leaders, and academics understand the complex relationship between paramedic system design and system performance, and, for the first time, provides empirical evidence upon which to make a conscious decision to adopt one system or the other.

## Introduction

As the paramedic profession matures and evolves, the journey toward professionalization has taken many different paths across the world. These paths have varied depending on each country's, and even local regions, socio-political environment. What is clear is that no two paramedic systems are the same. Yet, until recently, the age-old method of classifying paramedic systems and understanding their structure, function and performance, had not changed in 70 years [1].

In the past two decades, notable work by researchers such as Dick and O’Meara et. al. has sought to explore the divergence in structure and characteristics between paramedic systems. Their work has found that there is an immense difference between the way one system is structured and operates compared to another and that this is influenced by a myriad of complex micro and macro factors, including system leadership, professionalization status, and higher education to name a few [2-5].

A 2020 scoping review, published by this research team, identified two new systems of practice within the Anglo-American paramedic system. They were named the Directive and Professionally Autonomous paramedic systems [6]. Identifying what structures, characteristics, processes, and cultural factors contribute to system performance was the first part of our research series, work that was grounded by a conceptual framework [7]. We subsequently sought to define the two systems through expert consensus in a Delphi study [8]. This provided us with an understanding of the individual characteristics of each system, and importantly, validated the findings to ensure that there was confluence in the basis of our future research.

Identifying new systems of practice is important. However, understanding how each system performs when compared to prehospital quality indicators is equally important for establishing an argument for change toward a system that is better suited to the complex and changing health needs of its community.

Objective

The aim of this study was to compare paramedic perception of the Professionally Autonomous and Directive paramedic system performance using prehospital quality indicators.

## Materials and methods

Study design

A cross-sectional questionnaire was developed to measure and compare performance between the Professionally Autonomous and Directive paramedic systems.

Prehospital quality indicator construction

To assist with selecting the prehospital quality indicators for the survey, a set of criteria for inclusion and exclusion (Table 1) was created. These criteria helped to ensure the indicators used were supported by evidence, easily measurable, reflective of important aspects of care in paramedicine and finally were able to inform real change. The authors then reviewed contemporary research, including works by the Pre-Hospital Outcomes for Evidence-Based Evaluation [9-12] to help guide the development of the prehospital quality indicators list, with the final list (Table 2) being heavily influenced by a recent scoping review conducted by Pap et al. [13-14], which identified commonly utilized prehospital quality indicators across modern paramedic systems.

**Table 1:** Inclusion and exclusion criteria for the selection of prehospital quality indicators

Inclusion Criteria	Exclusion Criteria
(1) Prehospital quality indicator supported by scientific evidence or best practice. (2) Reflective of important aspects of care in contemporary paramedic settings (e.g., safety, effectiveness, patient-centeredness). (3) Actionable and capable of driving quality improvement.	(1) Lack of scientific evidence or best practices to support the quality indicator. (2) Overlap with other quality indicators or measures already in use. (2) Not feasible to collect or report on in a reliable or timely manner.

**Table 2:** List of prehospital quality indicators

Quality Indicator #	Indicator Type
Quality Indicator 1	Delivery of care in cardiac arrest
Quality Indicator 2	Delivery of care in acute coronary syndrome
Quality Indicator 3	Delivery of care in end of life
Quality Indicator 4	Delivery of care in trauma
Quality Indicator 5	Meaningful change in pain score
Quality Indicator 6	Clinical resources and resource management
Quality Indicator 7	Proportion of calls closed with telephone advice or managed without transport to the emergency department
Quality Indicator8	Proportion of decisions to leave a patient at the scene (‘hear and treat’ and ‘see and treat’)
Quality Indicator 9	Patient safety, satisfaction, and quality improvement
Quality Indicator 10	Employee welfare
Quality Indicator 11	Overall satisfaction

The authors originally planned to perform a retrospective analysis of system performance using the above-selected prehospital quality indicators; however, upon review of several quality reports published by paramedic systems, including London Ambulance Service and Austin Travis County EMS [15-16], it quickly became apparent that there was a lack of heterogeneity in what was publicly available.

To make up for this shortfall in openly available data, each indicator was adapted from established checklists into questions that measured paramedic perception of system performance and administered as part of an online questionnaire to informant paramedics.

Survey design

Data were collected using a questionnaire combining single-choice questions with matrix multiple-choice questions. The survey tool was designed to collect information in support of the research question using a constructivist approach and was designed and deployed through the Qualtrics© platform (Qualtrics, Provo, UT, USA). Distribution was primarily through social media and was open for responses over a 30-day period.

The first section of the questionnaire established which system type the paramedic worked in based on previously established definitions [8]. The second section of the questionnaire measured the perceived performance of each system using respondent knowledge of their own paramedic service. To minimize reviewer judgment and confirmation bias [17], we transformed criteria from checklists into data points and questions.

Throughout the questionnaire, respondents were asked to determine if a skill or action was considered “common practice.” Common practice was defined as something that is performed often and is considered normal practice.

A pilot was undertaken using experts to assess the functionality of the online survey platform as well as the clarity of questions. Additionally, the content validity index of the questions was tested using the expert panel to assess inter-rater agreement. An expert was defined as a (1) paramedic or physician in the field of paramedicine that held a senior leadership position within a medium to large Anglo-American paramedic system or (2) a senior academic who had studied and published on the topic of paramedic leadership, governance and/or paramedic system design. “Senior leadership position’ was defined as Director level or above, whilst a ‘medium size paramedic system’ was defined as more than 250 full-time equivalent salaried employees [8].

A total of 11 participants consented to participate in the pilot study. Each expert panel member evaluated each item (questions) for content validity on a four-point scale (1 = not relevant, 2 = somewhat relevant, 3 = quite relevant, 4 = highly relevant). The item level content validity index (I-CVI) (Appendices) was calculated as the number of experts giving a rating of either 3 or 4, divided by the number of experts [18,19]. Questions with an I-CVI rating below 0.80 were excluded from the final survey (appendix 1) [20]. Pilot data were not included in the analysis, although participants were invited to complete the final version of the survey. Following the review of the pilot data, nine questions were removed and two new questions related to patient registries were added owing to numerous requests from pilot participants.

Participants

Following the completion of a sample size calculation (Cochrans formula) [21], the target population was determined to be 375 respondents using a confidence level of 95%. The inclusion criteria for this survey were that participants were employed as a paramedic or an emergency medical technician working in an Anglo-American paramedic system in an English-speaking developed county. This included Australia, New Zealand, the United States, Canada, the United Kingdom, and the Republic of Ireland. Paramedics were excluded if they had a qualification less than basic life support or if they worked as a volunteer, as they were more likely to be considered a non-medically trained first responder such as a first aid-qualified driver.

Data analysis and statistical methods

Responses (yes, no, or unsure) were collected for 31 clinical questions across indicators one to 10. The two remaining questions covering indicator 11 utilised Likert scale responses where participants ranked their answers from 1 (strongly disagree) to 5 (strongly agree). Data are reported using descriptive statistics, including frequency (number and percentage). Associations between the Professionally Autonomous and Directive paramedic systems were determined using Pearson’s chi-squared (Χ2) or Wilcoxon rank-sum (Mann-Whitney U) tests where appropriate. Respondent satisfaction with leadership and clinical service provision was tested using a stepwise ordinal regression approach. A backward multivariate regression approach was utilised to determine the association with the dependent variable, with all demographic independent variables retained if p <0.2. All analysis was performed using Stata Version 15.1 (StataCorpLLC, College Station, TX, USA). Statistical significance was assigned if p<0.05.

Ethics

The study received human research ethics committee approval from the Monash University Human Research Ethics Committee and ascribed project number 32646. All participants provided written informed consent prior to commencing the online survey.

## Results

Demographics

There were 475 survey responses (Table 3), with 57.5% of respondents indicating that they worked in a Directive paramedic system, compared to 42.5% who worked in a Professionally Autonomous system. Most respondents were from the United States (35.6%), Canada (22.5%), Australia (18.1%) and the United Kingdom (18.1%). The survey identified a gender difference (Χ2=26.5, p<0.001) between systems, with 52.9% of Professionally Autonomous system participants being female as compared with only 30.7% in the Directive paramedic system group. The legal basis for practice varied amongst respondents however showed that those from a Directive paramedic system were more likely to be licensed (65.9%) as compared to respondents from the Professionally Autonomous paramedic system who were more likely to be registered (84.6%). Finally, level of practice also showed significant variance, with respondents from Directive paramedic systems indicating that they practised at an Emergency Medical Technician or Primary Care Paramedic scope (47.9%) compared to only 7.4% of paramedics from Professionally Autonomous paramedic systems whose most common level of practice was the Advanced Care Paramedic scope (39.6%).

**Table 3:** Respondent demographics

	Characteristic	Professionally Autonomous	Directive	Total
		n (%)	n (%)	n (%)
Age	18-29	83 (4.56%)	59 (21.61%)	142 (29.89%)
	30-39	67 (33.17%)	93 (34.06%)	160 (33.68%)
	40-49	35 (17.32%)	67 (24.54%)	102 (21.47%)
	50-59	15 (7.43%)	36 (13.17%)	51 (10.74%)
	60-69	1 (0.50%)	14 (5.12%)	15 (3.16%)
	Prefer not to say	1 (0.50%)	4 (1.47%)	5 (1.05%)
Gender	Male	94 (46.53%)	187 (68.50%)	281 (59.16%)
	Female	107 (52.97%)	84 (30.77%)	191 (40.21%)
	Non-binary/ diverse	1 (0.50%)	0 (0.00%)	1 (0.21%)
	Other	0 (0.00%)	2 (0.73%)	2 (0.73%)
Nationality	Australia	71 (35.15%)	15 (5.49%)	86 (18.11)
	New Zealand	19 (9.41%)	1 (0.37%)	20 (4.21%)
	Canada	30 (14.85%)	77 (28.21%)	107 (22.53%)
	United Kingdom	74 (36.63%)	12 (4.40%)	86 (18.11%)
	Republic of Ireland	5 (2.48%)	2 (0.73%)	7 (1.47%)
	United States	3 (1.49%)	166 (60.81%)	169 (35.58%)
Legal Basis for Practice	License	28 (13.86%)	178 (65.93%)	206 (43.64%)
	Registration (by regulator)	171 (84.65%)	64 (22.59%)	235 (49.47%)
	Authority to practice	2 (0.99%)	16 (5.93%)	18 (3.81%)
	Other	1 (0.50%)	15 (5.56%)	16 (3.39%)
Current Level of Practice	Emergency Medical Technician	3 (1.49%)	74 (27.11%)	77 (16.21%)
	Primary Care Paramedic	12 (5.94%)	57 (20.88%)	69 (14.53%)
	Advanced Care Paramedic	80 (39.60%)	50 (18.32%)	130 (27.37%)
	Specialist Paramedic	14 (6.93%)	6 (2.20%)	20 (4.21%)
	Extended Care Paramedic	3 (1.49%)	2 (0.73%)	5 (1.05%)
	Advanced Paramedic Practitioner	15 (7.43%)	7 (2.56%)	22 (4.63%)
	Critical Care Paramedic	18 (8.91%)	23 (8.42%)	41 (8.63%)
	Intensive Care Paramedic	13 (6.44%)	5 (1.83%)	18 (3.79%)
	Community Paramedic	7 (3.47%)	10 (3.66%)	17 (3.58%)
	Paramedic	31 (15.34%)	31 (11.35%)	62 (13.05%)
	Other	6 (2.97%)	8 (2.93%)	14 (2.95%)
Main Role	Clinician	165 (81.6%)	180 (65.9%)	345 (72.63%)
	Educator	12 (5.94%)	19 (6.96%)	31 (6.53%)
	Researcher	2 (0.99%)	7 (2.56%)	9 (1.89%)
	Supervisor, Manager or Director	19 (9.41%)	54 (19.78%)	73 (15.37%)
	Executive	1 (0.50%)	6 (2.20%)	7 (1.47%)
	Other	3 (1.49%)	7 (2.56%)	10 (2.11%)

Prehospital quality indicators

The questionnaire results section has been structured into prehospital quality indicator domains as viewed by respondents in the questionnaire (Table 4).

**Table 4:** Prehospital quality indicator results

Question		Autonomous System		Directive System			
	Response	(n)	%	(n)	%	Chi2	Pr
Q1 - In my paramedic service it is common practice that a caller requesting assistance for suspected/confirmed cardiac arrest is offered instructions (audio, or video if possible) in chest-compression-only CPR.	Yes	197	97.52%	187	68.50%		
	No	2	0.99%	83	30.40%	68.3634	0.000
	Unsure	3	1.49%	3	1.10%		
Q2 - In my paramedic service, it is common practice that a patient is identified to be in cardiac arrest by the emergency medical call-taker before the first resource arrives on the scene.	Yes	199	98.51%	190	69.60%		
	No	2	0.99%	82	30.04%	67.2895	0.000
	Unsure	1	0.50%	1	0.37%		
Q3 - My paramedic service contributes to a cardiac arrest registry.	Yes	163	80.69%	116	42.49%		
	No	12	5.94%	122	44.69%	90.6613	0.000
	Unsure	27	13.37%	35	12.82%		
Q4 - My paramedic service publishes publicly available data on patient survival to arrival at the hospital in an out-of-hospital cardiac arrest.	Yes	159	78.71%	61	22.34%		
	No	18	8.91%	157	57.51%	158.2329	0.000
	Unsure	25	12.38%	55	20.15%		
Q5 - My paramedic service has a documented clinical care pathway that details the care and transport it provides to patients with signs and/or symptoms suggestive of acute coronary syndrome.	Yes	197	97.52%	173	63.37%		
	No	4	1.98%	91	33.33%	78.7779	0.000
	Unsure	1	0.50%	9	3.30%		
Q6 - In my paramedic service it is common practice that every ambulance is equipped with a 12-lead electrocardiogram capable monitor/ defibrillator.	Yes	197	97.52%	155	56.78%		
	No	4	1.98%	116	42.49%	101.5339	0.000
	Unsure	1	0.50%	2	0.73%		
Q7 - In my paramedic service it is common practice that a patient with signs and/or symptoms suggestive of acute coronary syndrome has a 12-lead electrocardiogram acquired and interpreted by a clinician within 10 minutes of arrival on the scene.	Yes	193	95.54%	154	56.41%		
	No	6	2.97%	115	42.12%	94.2084	0.000
	Unsure	3	1.49%	4	1.47%		
Q8 - In my paramedic service it is common practice that, a patient presenting with signs and/or symptoms suggestive of acute coronary syndrome and normoxaemia (SpO2 >94%) is not administered supplementary oxygen.	Yes	197	97.52%	173	63.37%		
	No	3	1.49%	92	33.70%	79.7038	0.000
	Unsure	2	0.99%	8	2.93%		
Q9 - In my paramedic service it is common practice that, a patient with signs and/or symptoms suggestive of acute coronary syndrome is administered aspirin unless contraindicated.	Yes	200	99.01%	193	70.70%		
	No	0	0.00%	78	28.57%	69.0549	0.000
	Unsure	2	0.99%	2	0.73%		
Q10 - In my paramedic service it is common practice that, a patient with acute chest pain suggestive of acute coronary syndrome is administered analgesic agent(s) unless contraindicated.	Yes	198	98.02%	149	54.58%		
	No	3	1.49%	118	43.22%	111.6706	0.000
	Unsure	1	0.50%	6	2.20%		
Q11 - In my paramedic service it is common practice that, if transport time to a hospital capable of providing primary percutaneous coronary intervention is ≤30 minutes, a patient with ST-elevation myocardial infarction and within 12 hours of symptom onset is transported directly to that hospital.	Yes	192	95.05%	159	58.24%		
	No	7	3.47%	107	39.19%	83.6788	0.000
	Unsure	3	1.49%	7	2.56%		
Q12 - In my paramedic service it is common practice that, if transport time to a hospital capable of providing primary percutaneous coronary intervention is >30 minutes, a patient with STEMI, and within 12 hours of symptom onset receives prehospital fibrinolysis.	Yes	138	68.32%	50	18.32%		
	No	59	29.21%	206	75.46%	121.3796	0.000
	Unsure	5	2.48%	17	6.23%		
Q13 - My paramedic service has a documented clinical care pathway that details the delivery of end-of-life (palliative) care.	Yes	174	86.14%	90	32.97%		
	No	23	11.39%	170	62.27%	134.6422	
	Unsure	5	2.48%	13	4.76%		
Q14 - My paramedic service contributes to a trauma registry.	Yes	160	79.21%	102	37.36%		
	No	10	4.95%	129	47.25%	107.8661	0.000
	Unsure	32	15.84%	42	15.38%		
Q15 - In my paramedic service it is common practice that, a patient with a mechanism of injury and/or other signs/symptoms suggestive of pelvic fracture has a pelvic circumferential compression device applied.	Yes	197	97.52%	139	50.92%		
	No	3	1.49%	131	47.99%	124.6530	0.000
	Unsure	2	0.99%	3	1.10%		
Q16 - In my paramedic service it is common practice that, a patient with a recent (≤3 hours) traumatic injury resulting in ongoing hemorrhage and/or acute traumatic coagulopathy (indicated by a validated and pre-hospitally applicable prediction tool) receives tranexamic acid.	Yes	180	89.11%	106	38.83%		
	No	19	9.41%	161	58.97%	124.3344	0.000
	Unsure	3	1.49%	6	2.20%		
Q17 - In my paramedic service it is common practice that, a patient is correctly triaged and transported to an appropriate hospital as per the agreed trauma system protocol.	Yes	194	96.04%	171	62.64%		
	No	6	2.97%	99	36.26%	75.0857	0.000
	Unsure	2	0.99%	3	1.10%		
Q18 - In my paramedic service it is common practice that, an adult patient has their pain intensity measured using the 0-10 verbal numerical rating scale.	Yes	201	99.50%	182	66.67%		
	No	0	0.00%	89	32.60%	81.4838	0.000
	Unsure	1	0.50%	2	0.73%		
Q19 - In my paramedic service it is common practice that, a patient reporting mild (2-3/10), moderate (4-6/10) or severe (7-10/10) pain is administered analgesic agent(s), unless contraindicated or refused.	Yes	197	97.52%	137	50.18%		
	No	2	0.99%	128	46.89%	127.4082	0.000
	Unsure	3	1.49%	8	2.93%		
Q20 - My paramedic service has arrangements in place enabling paramedics to consult with a senior paramedic clinician via telephone when treating a patient.	Yes	180	89.11%	75	27.47%		
	No	20	9.90%	194	71.06%	178.7599	0.000
	Unsure	2	0.99%	4	1.47%		
Q21 - My paramedic service operates a secondary triage service (also known as a referral service or telephone triage service).	Yes	163	80.69%	56	20.51%		
	No	28	13.86%	207	75.82%	182.1274	0.000
	Unsure	11	5.45%	10	3.66%		
Q22 - In my paramedic service it is common practice that a patient who meets service-defined treat-and-discharge or treat-and-refer criteria is not transported.	Yes	183	90.59%	90	32.97%		
	No	17	8.42%	178	65.20%	158.8313	0.000
	Unsure	2	0.99%	5	1.83%		
Q23 - My paramedic service has a policy/ clinical practice guideline that describes the treat-and-refer arrangements for patients not conveyed to a health care facility.	Yes	177	87.62%	81	29.67%		
	No	19	9.41%	185	67.77%	163.9262	0.000
	Unsure	6	2.97%	7	2.56%		
Q24 - In my paramedic service it is common practice that, a patient who is not conveyed to a healthcare facility has been risk-assessed for the likelihood of deterioration.	Yes	184	91.09%	118	43.22%		
	No	12	5.94%	142	52.01%	118.7843	0.000
	Unsure	6	2.97%	13	4.76%		
Q25 - In my paramedic service it is common practice that, a patient who is not conveyed to a healthcare facility receives a clinical follow-up within 48 hours.	Yes	116	57.43%	20	7.33%		
	No	74	36.63%	238	87.18%	146.9743	0.000
	Unsure	12	5.94%	15	5.49%		
Q26 - My paramedic service has a dedicated patient safety reporting system.	Yes	178	88.12%	99	36.26%		
	No	17	8.42%	152	55.68%	130.4311	0.000
	Unsure	7	3.47%	22	8.06%		
Q27 - My paramedic service collects and analyses data pertaining to patient experience and satisfaction for the purpose of quality improvement.	Yes	165	81.68%	95	34.80%		
	No	21	10.40%	157	57.51%	115.3976	0.000
	Unsure	16	7.92%	21	7.69%		
Q28 - My paramedic service operates a quality improvement program that includes quality assessment/ measurement, control, and improvement.	Yes	179	88.61%	137	50.18%		
	No	13	6.44%	116	42.49%	82.3839	0.000
	Unsure	10	4.95%	20	7.33%		
Q29 - My paramedic service schedules paramedics to work shifts 12 hours or shorter in duration.	Yes	186	92.08%	130	47.62%		
	No	15	7.43%	141	51.65%	103.7316	0.000
	Unsure	1	0.50%	2	0.73%		
Q30 - My paramedic service provides mental health programs, including critical incident stress response and/ or pre-incident preparedness training, to its paramedics.	Yes	182	90.10%	131	47.99%		
	No	17	8.42%	135	49.45%	92.9799	0.000
	Unsure	3	1.49%	7	2.56%		
Q31 - My paramedic service collects and analyses data pertaining to staff experience and satisfaction for the purpose of quality improvement.	Yes	167	82.67%	77	28.21%		
	No	29	14.36%	183	67.03%	140.1625	0.000
	Unsure	6	2.97%	13	4.76%		

Prehospital quality indicator 1: cardiac arrest (Q1-4)

Respondents in Professionally Autonomous services indicated that callers requesting assistance for a suspected cardiac arrest were offered instructions in chest-compression-only cardio-pulmonary resuscitation 97.5% of the time compared to 68.5% in Directive systems. Similarly, 98.5% of respondents in Professionally Autonomous services indicated it is common practice that a patient will be identified to be in cardiac arrest by the emergency medical call-taker before the first resource arrives on the scene compared to 69.6% of respondents in Directive systems. Significantly more (80.6%) respondents from a Professionally Autonomous paramedic system reported their organization contributed to a cardiac arrest registry, compared to 42.4% of respondents from a Directive paramedic system.

Prehospital quality indicator 2: acute coronary syndrome (Q5-12)

Almost all respondents (97.5%) from Professionally Autonomous services stated they had a documented clinical care pathway that details the care and transport provided to patients with signs and symptoms suggestive of acute coronary syndrome. Similarly, as common, 97.5% of Professionally Autonomous ambulances are equipped with a 12-lead ECG-capable monitor/defibrillator compared to only 56.7% of Directive service ambulances. With respect to medication administration, respondents indicated that patients presenting with signs and symptoms suggestive of acute coronary syndrome and normoxaemia (SpO2 >94%) were not administered supplementary oxygen in 97.5% of events within Professionally Autonomous systems, compared to 63.3% of respondents from Directive paramedic systems. Aspirin administration in acute coronary syndrome was reported by 99% of respondents from a Professionally Autonomous paramedic system and 98% receive analgesia as part of their treatment, compared to 70.7% and 54.6%, respectively, of Directive system clinicians.

Prehospital quality indicator 3: delivery of care in end of life (Q13)

Respondents from Professionally Autonomous paramedic systems indicated that they had higher access to clinical care pathways that detail the delivery of end-of-life (palliative) care (86.1%) compared to 32.9% of Directive paramedic service respondents.

Prehospital quality indicator 4: delivery of care in trauma (Q14-17)

As an initial measure of a service’s research and development, 79.2% of respondents from Professionally Autonomous services stated their service participates in a trauma registry while 37.3% from Directive services stated the same. There was a significant difference between service models in the delivery of tranexamic acid, where a patient with recent (≤3 hours) traumatic injury resulting in ongoing hemorrhage or acute traumatic coagulopathy receives the medication in 89.1% of Professionally service responses compared to 38.7% of Directive service responses.

Prehospital quality indicator 5: meaningful change in pain score (Q18-19)

An overwhelming number of respondents (99.5%) from Professionally Autonomous systems stated it is common practice that an adult patient has their pain intensity measured using the 0-10 verbal numerical rating scale, compared to 66.6% of respondents from a Directive system. Moreover, whilst 97.5% of respondents from Professionally Autonomous services reported that their paramedic service administers analgesic agent(s) to patients reporting mild (2-3/10), moderate (4-6/10) or severe (7-10/10) pain unless contraindicated or refused, only 50.1% of respondents from the Directive services did the same.

Prehospital quality indicator 6: clinical resource and resource management (Q20)

Respondents in Professionally Autonomous systems reported higher rates of access, enabling them to consult with a senior paramedic clinician via telephone when treating a patient (89.1%) compared to 27.4% of respondents in the Directive group.

Prehospital quality indicator 7: proportion of calls closed with telephone advice or managed without transport to the emergency department(Q21)

Respondents in the Professionally Autonomous group shared that they operate a secondary triage service in 80.6% of cases compared to 27.4% of respondents in the Directive group. This represents an approximately 60% difference between both groups, which was among the most significant across all survey questions.

Prehospital quality indicator 8: proportion of decisions to leave a patient at the scene (‘hear and treat’ and ‘see and treat’) (Q22-25)

When asked whether it is common practice that a patient who meets service-defined treat-and-discharge or treat-and-refer criteria is not transported in their paramedic services, 90.5% of respondents from Professionally Autonomous services stated “yes” compared to 32.9% from Directive services. In alignment with the non-transport pathway availability, 87.6% of Professionally Autonomous service respondents had a policy/clinical practice guideline that describes the treat-and-refer arrangements for patients not conveyed to a health care facility, as compared to 29.6% of Directive service respondents. Finally, patients who are not conveyed to a healthcare facility receive a clinical follow-up within 48 hours in 57.4% of Professionally Autonomous system responses while this occurs in 7.3% of all Directive system responses.

Prehospital quality indicator 9: patient safety, satisfaction and quality improvement (Q26-28)

Clinicians from Professionally Autonomous systems reported their paramedic service has a dedicated patient safety reporting system in 88.1% of responses. Likewise, most respondents from Professionally Autonomous systems (81.6%) indicated that their paramedic service collects and analyses data pertaining to patient experience and satisfaction for the purpose of quality improvement. Lastly, a far higher proportion of Professionally Autonomous system paramedics (88.6%) reported they had a quality improvement program that includes quality assessment/measurement, control and improvement in comparison to the Directive system cohort (50.1%).

Prehospital quality indicator 10: employee welfare (Q29-31)

Amongst participants from Directive paramedic services, 51.6% shared that their employer schedules paramedics to work shifts 12 hours or longer in duration with only 7.4% of Professionally Autonomous systems doing the same. Professionally Autonomous cohort respondents (90.1%) shared that their paramedic service provides mental health programs to its paramedics compared to 47.9% in Directive services.

Prehospital quality indicator 11: overall satisfaction

Two additional survey questions were dedicated to overall employee satisfaction (Table 5). Satisfaction was determined to be higher in the Professionally Autonomous cohort. Overall satisfaction with their services’ clinical care standard followed a similar pattern, with 87.1% of participants in a Professionally Autonomous system stating they were “satisfied” or “very satisfied” with the care they deliver compared to 39.5% of Directive system respondents. There was a significant difference between group responses, with Professionally Autonomous respondents more satisfied with leadership (z=9.81, p<0.001) and care delivery (z=11.25, p<0.001) within their service.

**Table 5:** Satisfaction levels in autonomous and directive systems, including the two-sample Wilcoxon rank-sum (Mann-Whitney) test

	Characteristic	Autonomous	Directive	Total
		n (%)	n (%)	n (%)
Q32 - Overall satisfaction with the senior leadership team	Very Satisfied	27 (13.17%)	15 (5.49%)	42 (8.84%)
	Satisfied	115 (56.93%)	52 (19.05%)	167 (35.16%)
	Neutral	19 (9.41%)	32 (11.72%)	51 (10.74%)
	Dissatisfied	31 (15.35%)	107 (39.19%)	138 (29.05%)
	Very dissatisfied	10 (4.95%)	67 (24.54%)	77 (16.21%)
Q33 - Overall satisfaction with service clinical care standard	Very Satisfied	67 (33.17%)	21 (7.69%)	88 (18.53%)
	Satisfied	109 (53.96%)	87 (31.87%)	196 (41.26%)
	Neutral	13 (6.44%)	30 (10.99%)	43 (9.05%)
	Dissatisfied	13 (6.44%)	86 (31.50%)	99 (20.84%)
	Very dissatisfied	0 (0.00%)	49 (17.95%)	49 (10.32%)

The ordinal regression data are presented in Tables 6, 7. Multivariate associations were significant for service type (p<0.001), country (p<0.001) and age group (p=0.001) for satisfaction in leadership (Χ25 =142.77, p<0.001, R2=0.103). While the associations were significant for service type (p<0.001), country (p<0.001), years of practice (p<0.001) and legal basis of practice (p=0.002) for satisfaction with service provision (Χ24 =203.26, p<0.001, R2=0.149). Gender and level of practice were removed in both models.

**Table 6:** Respondents' satisfaction with senior leadership. Stepwise ordinal regression results

Demographic factor	Coefficient	95% Confidence Interval	P-value
Service Type	-1.416	-1.878, -0.954	<0.001
Country	-0.335	-0.489, -0.181	<0.001
Gender	0.236	-0.099, 0.571	0.169
Age Group	0.279	0.117, 0.442	0.001
Role	0.088	-0.026, 0.202	0.132

**Table 7:** Respondents' satisfaction with their services' clinical care standards. Stepwise ordinal regression results

Demographic factor	Coefficient	95% Confidence Interval	P value
Service Type	-1.768	-2.263, -1.272	<0.001
Country	-0.328	-0.490, -0.165	<0.001
Years of practice	0.054	0.035, 0.073	<0.001
Legal practice basis	0.488	0.175, 0.800	0.002

## Discussion

The results of this study portray paramedic perception of the performance of the two systems of practice under investigation and have highlighted significant disparity with respect to the perceived performance of each system.

Staff and demographics

The respondent demographics (Table 2) are a vital step in validating the results of a previously published Delphi study [8] and confirms several previously held assumptions. First, it validates that the Professionally Autonomous paramedic system is largely found in Australia, New Zealand and the UK, with the majority of the workforce indicating they hold a registration issued by a regulatory board and practice at an advanced clinical level, i.e. Specialist Paramedic. Second, it confirms that most respondents who identified as working within a Directive paramedic system are overwhelmingly located in the US and Canada, are licensed rather than registered and practice at a basic life support level (emergency medical technician and primary care paramedic) reflecting the significant differences in education between the two systems [22-24]. The third point worth noting is that those who identified themselves as operating within a Professionally Autonomous paramedic system tended to be younger, a fact that has been substantiated by the yearly demographics report of the Council of Ambulance Authorities [25]. Finally, the results confirm gender balance between the two systems was consistent with previous evidence indicating Professionally Autonomous systems have made great strides in achieving gender equity in the workforce [25].

Perceptions of care standards

This survey assessed five common clinical domains as a means of assessing respondents’ perception of the quality of care provided within their system. With respect to out-of-hospital cardiac arrest systems identified to be Directive in nature, they were less likely to offer instruction to callers in chest-compression-only cardiopulmonary resuscitation and to identify a patient to be in cardiac arrest prior to the arrival of the first ambulance. This is surprising given the strong advocacy by the US-based Global Resuscitation Alliance [26] whose aim is to help to improve survival in out-of-hospital cardiac arrests through their 10-step community-based program. One hypothesis may be that the Global Resuscitation Alliance is more impactful in larger jurisdictional systems such as those found in Australia, New Zealand, the United Kingdom and parts of Canada when compared to the United States, a country that lacks a nationally standardized system or scope of practice [27]. As such, the United States has thousands of paramedic systems that must conform to their respective state's and local jurisdiction's specific legislation, regulations, medical protocols, scopes of practice, standards of care, policies, procedures and requirements [27]. Similarly, it was surprising that Directive respondents, mostly based in the United States, indicated their system did not contribute to a cardiac arrest registry given the availability of a national free-to-access Resuscitation Outcomes Consortium Epistry [28]. Similar national registries exist in the United Kingdom, Australia and New Zealand where respondents indicated better uptake by their systems [29,30].

Care of acute coronary syndrome patients showed a similar difference with far fewer respondents from Directive systems indicating they were equipped with a 12-lead electrocardiogram capable monitor/defibrillator, which also directly correlated with their ability to perform a 12-lead ECG on patients presenting with signs and symptoms of acute coronary syndrome as well as providing a referral to a centre capable of providing primary percutaneous coronary intervention. This is likely linked back to the reduced scope of practice driven by a workforce that is predominantly trained in a technical institute. Cash et al. report that 86% of the 790 education programs in the US award a certificate, with the remaining 14% awarding either a diploma, associate degree or bachelor’s degree [23]. This contrasts with the Professionally Autonomous paramedic system where a degree to entry practice is mandatory for all new paramedic applicants resulting in the baseline qualification of advanced care paramedic (or equivalent) [22,31,32]. The same drivers likely influence the results related to the administration of analgesia to patients experiencing pain and the various trauma-related interventions.

In contrast, in what is considered a truly novel approach in expanding the paramedic-led delivery of healthcare beyond emergency health response, respondents from the Professionally Autonomous system reported that they had greater access to care pathways for end-of-life patients (86.1%). However, in balancing the discussion, the utilisation of paramedics within palliative care contexts is not without challenges. In Australia, for example, higher transport rates have been reported, with approximately three-quarters of the 4348 palliative care patients who contacted a state ambulance service in a 12-month period being conveyed to a hospital [33]. The core of the issue may lie in a lack of confidence and competence in managing palliative patients due to a perceived aperture in paramedic education across both systems [34].

Clinical advice and consultation

Similarly, many respondents in the Professionally Autonomous paramedic system (89.1%) indicated they had access to a paramedic-led clinical advice and consultation service with only 27.4% of respondents employed in a Directive system indicating they had access to the same resource. A recent study by Armour et al. [35], in which paramedic-led telehealth consultations were studied in British Columbia, Canada, noted that paramedics felt that their peers were uniquely suited to provide clinical consultations and advice in a way physicians were not based on their lived experience of out-of-hospital care. It's likely that the professionalization and self-regulation of paramedics combined with an undergraduate and postgraduate education have helped Professionally Autonomous paramedic services recognise the role paramedics have to play in delivering teleconsultations. Furthermore, with no uniform requirements for paramedics in the United States to undertake a higher education degree in order to enter practice, an argument for the irrelevance of the physician-led clinical advice model is hard to make. A question worth asking is whether the concept of medical direction has a role to play in hindering the inconsistent education of paramedic service providers in North America.

Virtual and integrated care

Comparisons between the Professionally Autonomous and Directive systems indicated that Professionally Autonomous paramedic systems offered a higher rate of telehealth (virtual care) services (80.6% vs 20.5%) as well as provided their workforce with a suite of non-conveyance options, including ‘hear and treat’ and ‘see and treat’ (90.5% vs 32.9%). Additionally, these same systems provided a higher rate of patient follow-up, a service directly correlated to the higher number of patients discharged by paramedics without further conveyance. There are many enablers that contribute to the successful delivery of telehealth services and alternative care pathways, most important of which is a paramedic system that is highly integrated into the local health network in which there is an equal funding model for conveyance vs. non-conveyance decisions [36]. Funding is a key barrier in Directive paramedic systems, particularly in the United States where paramedic regulations have historically only allowed payment for emergency ground ambulance services when individuals are transported to hospital [37]. Most patients who call an ambulance are therefore only transported to a hospital emergency department, even when a lower-acuity destination or no need for transportation may more appropriately meet an individual’s clinical care needs [38]. However, this model is now starting to shift, with the recent introduction of the Emergency Triage, Treat, and Transport innovation program by the US Government [37]. This program aims to change the funding model for paramedic systems away from incentivising conveyance to a hospital toward equal funding access for patients who are either (1) conveyed to alternate care centres, such as urgent primary care centres, (2) discharged with care and advice at home or (3) assessed using a telehealth model of care. This radical shift in policy is now transforming the way in which patients within Directive paramedic systems receive care with hundreds of providers already switching to telehealth models of care [39].

Clinical governance functions

One of the commonly touted benefits of medical direction in paramedicine is the ability to help integrate public safety-facing systems better into the healthcare system through governance functions provided by Medical Directors [40]. Yet, this survey identified that more than half of respondents who practice in a Directive paramedic system do not have a patient safety reporting system (55.6%) or quality improvement program (57.5%) within their service. A stark contrast to the Professionally Autonomous paramedic system, which is typically more fully integrated into the health care network and whose respondents indicated above 80% inclusion of the above governance functions. These results validate the recent findings of a literature review exploring the relationship between medical oversight and clinical governance, which concluded that there was little high-quality evidence supporting the effectiveness of “medical direction” model as a proxy for clinical quality and safety in paramedic services. Rather, clinical governance is more effective when modelled as a systems approach with shared responsibility for quality and safety [5].

Employee welfare initiatives

Directive paramedic systems again rated poorly with respect to employee welfare initiatives with over half of the respondents surveyed (51.6%), indicating they performed shifts greater than 12 hours in duration. The literature on this topic is highly developed and notes that for outcomes considered critical or important to paramedics, shifts <12 hours in duration are more favourable than shifts ≥12 hours [41,42]. Even more concerning was the absence of mental health support for paramedics in some systems, with 49.4% of respondents working in a Directive paramedic system noting they had no access to these supports, in contrast to 92.0% of paramedics in Professionally Autonomous paramedic systems identified that they did. There is an abundance of literature linking sleep, shift work and mental health supports together when caring for employee welfare [43,44], and the evidence overwhelmingly supports a reduction in depression, anxiety and improved well-being when employee welfare initiatives are implemented and utilized [45].

Limitations

This survey carries the risk of response bias from participants who seek to both exaggerate and/or understate the performance of their system [46]. Second, there can be a difference between perceptions of performance and performance itself, and this can lead to perception bias as a factor in the results of this study. This was addressed in the design of the questionnaire by ensuring that questions were not anchored to any system identifiers, however, ultimately the indicators are based on actions rather than outcomes. Third, the recruitment of participants through social media led to the presence of selection bias in the recruitment strategy. Fourth, the exclusion of volunteers as participants may have resulted in selection bias. Volunteers were excluded as participants, as there is often a lack of consistency in the definition and role of "paramedic" worldwide, and many volunteers are not medically trained first responders. Although we acknowledge that there are exceptions to this, we believed that excluding volunteers would lead to more accurate and clearer data for our research. Finally, whilst the demographic results have helped to validate previous definitions of the two systems, we found a natural cross-over in demographic information. For example, some participants who identified working in a Professionally Autonomous system indicate they are licensed rather than registered. Whilst human error can potentially account for some results, these results have also helped us to validate a previously held belief that many systems are hybrid in nature, a factor that was not accounted for as part of the questionnaire process.

## Conclusions

This research is a vital step in helping paramedics and leaders understand the complex relationship between paramedic system design and system performance, and for the first time, provides the evidence upon which to base an argument for change. The perceptions of paramedics across 11 prehospital quality indicators provide evidence indicating the Professionally Autonomous paramedic system outperforms the Directive system in all areas that were studied. Future research needs to explore a knowledge-to-action framework that supports systems to transition from a Directive model of care to that of the better performing Professionally Autonomous paramedic system as well as the development of consistent reporting frameworks for comparison and analysis of system performance.

## Appendices

**Table 8:** Item-level content validity index results

Item number	No. of respondents	Experts in Agreement	Item-level Content Validity Index	Item number	No. of respondents	Experts in Agreement	Item-level Content Validity Index
1	11	9	0.82	21	11	9	0.82
2	11	9	0.82	22	11	10	0.91
3	11	6	0.54	23	11	9	0.82
4	11	5	0.45	24	11	9	0.82
5	11	7	0.64	25	11	7	0.64
6	11	6	0.54	26	11	11	1.00
7	11	10	0.91	27	11	8	0.73
8	11	10	0.91	28	11	11	1.00
9	11	9	0.82	29	11	11	1.00
10	11	9	0.82	30	11	11	1.00
11	11	10	0.91	31	11	11	1.00
12	11	9	0.82	32	11	11	1.00
13	11	8	0.73	33	11	11	1.00
14	11	8	0.73	34	11	11	1.00
15	11	9	0.82	35	11	11	1.00
16	11	9	0.82	36	11	10	0.91
17	11	11	1.00	37	11	11	1.00
18	11	11	1.00	38	11	11	1.00
19	11	8	0.73	39	11	9	0.82
20	11	9	0.82	40	11	11	1.00
Average Item-level content validity index results							0.85
